# Measuring lineup fairness from eyewitness identification data using a multinomial processing tree model

**DOI:** 10.1038/s41598-023-33101-6

**Published:** 2023-04-18

**Authors:** Nicola Marie Menne, Kristina Winter, Raoul Bell, Axel Buchner

**Affiliations:** Department of Experimental Psychology, Heinrich Heine University Düsseldorf, Düsseldorf, Germany

**Keywords:** Psychology, Human behaviour

## Abstract

The mock-witness task is typically used to evaluate the fairness of lineups. However, the validity of this task has been questioned because there are substantial differences between the tasks for mock witnesses and eyewitnesses. Unlike eyewitnesses, mock witnesses must select a person from the lineup and are alerted to the fact that one lineup member might stand out from the others. It therefore seems desirable to base conclusions about lineup fairness directly on eyewitness data rather than on mock-witness data. To test the importance of direct measurements of biased suspect selection in eyewitness identification decisions, we assessed the fairness of lineups containing either morphed or non-morphed fillers using both mock witnesses and eyewitnesses. We used Tredoux’s *E* and the proportion of suspect selections to measure lineup fairness from mock-witness choices and the two-high threshold eyewitness identification model to measure the biased selection of the suspects directly from eyewitness identification decisions. Results obtained in the mock-witness task and the model-based analysis of data obtained in the eyewitness task converged in showing that simultaneous lineups with morphed fillers were significantly more unfair than simultaneous lineups with non-morphed fillers. However, mock-witness and eyewitness data converged only when the eyewitness task mimicked the mock-witness task by including pre-lineup instructions that (1) discouraged eyewitnesses to reject the lineups and (2) alerted eyewitnesses that a photograph might stand out from the other photographs in the lineup. When a typical eyewitness task was created by removing these two features from the pre-lineup instructions, the morphed fillers no longer lead to unfair lineups. These findings highlight the differences in the cognitive processes of mock witnesses and eyewitnesses and they demonstrate the importance of measuring lineup fairness directly from eyewitness identification decisions rather than indirectly using the mock-witness task.

## Introduction

Mistaken eyewitness identification is a consistent and leading cause of wrongful convictions. In the United States, eyewitness misidentifications have contributed to 70 % of the more than 375 wrongful convictions uncovered by DNA-based exonerations^1^. One reason for wrongful convictions is that unfair lineups increase the likelihood of misidentifications of innocent suspects^2,3^. A lineup is considered fair when all fillers (distractors who are known to be innocent) serve as plausible alternatives to the suspect in the lineup such that there is no way to distinguish the suspect from the other lineup members without relying on memory for the culprit. Fair lineups provide protection of the innocent suspect because good fillers siphon misidentifications away from the innocent suspect^4,5^. This protective mechanism is absent in unfair lineups in which the suspect stands out from the other lineup members based on physical appearance or other distinct characteristics of the suspect’s photograph^4,6^. It is clear from prior studies that unfair lineups dramatically increase the risk of mistakenly identifying the suspect in comparison to fair lineups^2,3,7,8^. For this reason, it is important to understand the numerous factors that can influence lineup fairness. However, progress will only be made when the fairness of a lineup is measured in a valid way.

Eyewitness researchers have typically used the mock-witness task^9^ to assess lineup fairness^10^. In this task, persons who did not witness the crime—so-called mock witnesses—are asked to view the lineup and to choose the lineup member they believe to be the police suspect. One possibility is that mock witnesses are provided with the witness’s description of the culprit as the basis for their choices [e.g.,^11,12^]. Alternatively, mock witnesses are not provided with any additional information other than the indication that the suspect might stand out from the other lineup members; armed with this information, mock witnesses are simply asked to indicate who they think the suspect is [e.g.,^13,14^]. This most basic evaluation of lineup fairness can be used to investigate whether there are cues that make the suspect stand out from the other lineup members that are unrelated to the facial appearance of the culprit^15^. For example, in photo lineups the facial photograph of the suspect may stem from a different source (e.g., social media) than the photographs of the fillers that may be taken from special databases^16^. Sometimes, the photographs of the fillers may even be digitally manipulated^8,17^. Therefore, a simple inspection of the characteristics of the photographs such as brightness, contrast, color balance or softness could reveal who the suspect is (more on this below). Given that mock witnesses have not seen the face of the culprit, they cannot make an identification that is based on memory. Instead, they have to rely on inferences that are either informed by a description of the culprit or based on other clues available in the lineup. A lineup is fair if the mock-witness choices are evenly distributed among the lineup members (in a six-person lineup, each lineup member, including the suspect, should be selected by 1 ÷ 6 of the mock witnesses). A lineup is unfair if disproportionately many mock witnesses select the suspect^9^. Based on the choices of mock witnesses, several formal measures of lineup fairness can be computed. These measures reflect either the effective lineup size (as opposed to the nominal lineup size) or the bias with which the suspect is selected^15,18^. Effective lineup-size measures indicate the number of lineup members that could plausibly be considered as the culprit. One of the most popular effective lineup-size measures is Tredoux’s* E*^19^. The proportion of suspect selections^9^ is a popular measure of the bias with which the suspect is selected. This measure reflects the extent to which the suspect stands out from the other lineup members.

The mock-witness task was originally developed to measure lineup fairness in real criminal cases in which the suspect’s guilt is unknown to the police, not for measuring lineup fairness in laboratory experiments^20^. Nevertheless, it has become increasingly common in experimental research to rely on the mock-witness task^10^ as it provides a seemingly straightforward solution to the problem of how to assess the fairness of lineups. However, the validity of the mock-witness task has been criticized on the grounds that there are substantial differences between the tasks of mock witnesses and eyewitnesses^10,15,21^.

First, mock witnesses are typically encouraged or even forced to choose one of the lineup members while lineup rejections are discouraged or even prevented, respectively. If participants are discouraged from rejecting the lineup but ignore or defy these instructions, their data are excluded from analysis [e.g.,^22^]. However, in order to avoid this loss of data, mock witnesses are typically denied the option to reject the lineup. Instead, mock witnesses are usually forced to guess who the suspect is [e.g.,^9,14^]. In contrast, eyewitnesses are encouraged to reject the lineup if they are unsure as to whether or not the culprit is in the lineup. More specifically, eyewitnesses are typically given two-sided pre-lineup instructions that emphasize the fact that it is equally important to select the culprit in culprit-present lineups and to reject culprit-absent lineups [e.g.,^23–25^]. This is also the procedure recommended by several guidelines for how lineups should be conducted^26–28^. Two-sided instructions decrease the probability of selecting one of the lineup members based on guessing, which is highly desirable in eyewitness tasks because the reduction of guessing-based selections reduces false identifications of innocent suspects that could lead to wrongful convictions^29–32^.

Second, the task of a mock witness differs necessarily from that of an eyewitness. Given that mock witnesses have not seen the face of the culprit, they cannot make a memory-based decision but have to perform a non-memory-based comparison of the faces in the lineup. Unlike eyewitnesses, mock witnesses are thus alerted to the fact that one person might stand out from the other lineup members. When using a description-based mock-witness task, participants are typically asked to choose the person who best fits the culprit’s description which implies that the description fits one person better than the others [e.g.,^11^]. When no description is presented, mock witnesses are explicitly told to choose the person who looks most distinctive or stands out from the other lineup members [e.g.,^13^]. Both types of instructions can be expected to encourage non-memory-based comparisons among the lineup members which may make participants sensitive to unfairness cues, possibly to the degree to which participants notice cues they would not have noticed otherwise. When participants are not provided with a description of the culprit’s face, it is impossible to search for the culprit in the lineup and the only remaining strategy is to carefully compare the photographs in the lineup to identify the face that stands out. This is markedly different from the memory-based identification task of eyewitnesses who have to match each lineup member to their memory representation of the culprit in order to decide whether or not one person represents the culprit^33^. Any features that are unrelated to the identity of the culprit such as brightness, contrast, color balance and softness of the photographs are irrelevant to this task and may be thus ignored by the eyewitnesses. Given these striking differences between the mock-witness task and the eyewitness task, the processes underlying the observed behavior may well differ between mock witnesses and eyewitnesses. It is thus unclear whether the mock-witness task can be used to draw valid conclusions about eyewitness identification decisions.

Fortunately, it is not necessary to rely on the mock-witness task to arrive at measures of lineup fairness. This is so because a valid measurement model is available for estimating biased suspect selection in unfair lineups directly from eyewitness data: the two-high threshold (2-HT) eyewitness identification model^32,34^. This model belongs to the class of multinomial processing tree (MPT) models, a family of models for estimating the probability of latent processes from categorical data^35,36^. For an overview of the MPT modeling approach, we recommend the very useful tutorial by Schmidt et al.^37^. Based on the full range of data categories observed in the eyewitness task (that is, suspect identifications, filler identifications and lineup rejections in both culprit-present and culprit-absent lineups), the model provides measures of the latent processes underlying eyewitness identification decisions. Specifically, the set of processes measured by the 2-HT eyewitness identification model comprises the detection of culprit presence and absence, the selection of a lineup member based on guessing and, most importantly in the present context, the process of biased suspect selection. The process of biased suspect selection will play a central role here because it reflects the process of selecting a suspect that stands out from the fillers in unfair lineups, as validation studies have shown^32,34^.

**Figure 1:**
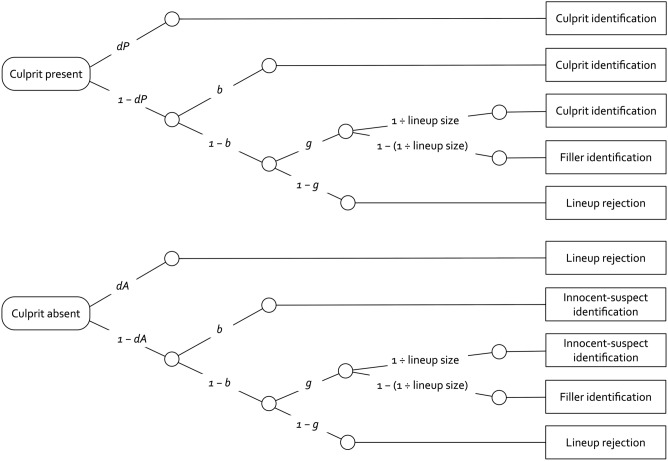
The 2-HT eyewitness identification model^32,34^. The rounded rectangles on the left represent the lineup types presented to the participants: culprit-present and culprit-absent lineups. The parameters attached to the branches of the trees denote transition probabilities of the latent cognitive processes postulated by the model (*dP:* probability of detecting the presence of the culprit; *b:* probability of biased selection of the suspect; *g:* probability of guessing-based selection among the lineup members; *dA:* probability of detecting the absence of the culprit). *Lineup size* represents the number of persons in the lineup. The rectangles on the right side show the categories of the observable responses.

A graphical illustration of the 2-HT eyewitness identification model is shown in Fig. 1. The model tree in the upper half of Fig. 1 illustrates the latent processes underlying eyewitness identification decisions from lineups in which the culprit is present. A culprit is detected with probability *dP* (for detection of the presence of the culprit). If participants do not detect the culprit, which occurs with probability 1 − *dP,* then two types of non-detection-based processes can still lead to the correct identification of the culprit in lineups with the culprit present. First, and most importantly for the present purposes, participants may select the suspect without relying on memory if the suspect stands out from the fillers. This process of biased suspect selection in unfair lineups is represented by parameter *b*. Second, in case of no biased selection of the suspect, which occurs with probability 1 − *b,* participants can still select one of the lineup members based on guessing with probability *g* (for guessing-based selection). In this case, participants will either pick out the suspect with a probability equal to 1 ÷ lineup size (approximately 0.16667 in the present case of six lineup members) or they will select one of the fillers with the complementary probability 1 − (1 ÷ lineup size)*.* Guessing-based selection of one of the lineup members does not occur with probability 1 − *g*, in which case participants reject the lineup by not making an identification.

The model tree in the lower half of Fig. 1 refers to lineups from which the culprit is absent. Participants may correctly detect the absence of the culprit with probability *dA* (for detection of the absence of the culprit), resulting in a correct lineup rejection. If culprit-absence detection fails, which occurs with probability 1 − *dA*, the same non-detection-based biased and guessing-based selection processes occur as in culprit-present lineups: With probability *b,* the innocent suspect may stand out from the other lineup members and prompt participants to incorrectly select the innocent suspect. No biased selection occurs with probability 1 − *b*. In this case participants may still select a lineup member based on guessing with probability *g*. In culprit-absent lineups, this leads participants either to incorrectly pick out the innocent suspect (with probability 1 ÷ lineup size) or to select one of the fillers (with probability 1 − [1 ÷ lineup size])*.* Alternatively, participants may not select a lineup member based on guessing with probability 1 − *g,* which results in a correct rejection of the lineup in culprit-absent lineups.

The 2-HT eyewitness identification model has been extensively validated using novel experiments designed specifically for the purpose of testing the model’s validity^32^ and by fitting the model to published data obtained in various laboratories^34^. Both approaches support the validity of the model by demonstrating that all parameters predictably reflect experimental manipulations of the processes they were designed to measure. A brief overview of the validation results for the biased-suspect-selection parameter *b* seems in order because this parameter is of central importance to the present study. Parameter *b* has been shown to sensitively reflect the unfairness of a lineup in which the suspect’s face stood out from the fillers’ faces because it was the only face without large birthmarks^32^. In addition, the biased-suspect-selection parameter *b* has been shown to be larger in unfair lineups with low suspect-filler similarity than in fair lineups with high suspect-filler similarity; parameter *b* has also been shown to be larger when the suspect’s face stood out from the fillers due to distinctive facial features such as scars, bruisings, nose piercings and tattoos than when the suspect’s face did not stand out^34^.

In the experiments reported here, we measured the fairness of lineups containing either morphed or non-morphed photographs of fillers (hereinafter referred to as morphed and non-morphed lineups). This morphing manipulation is of applied relevance. Assembling lineups is often a challenging task because pertinent databases often do not provide enough facial photographs that match the description of the culprit^3,38^. To solve this problem, face-morphing software can be used to increase the selection of faces that can be used in the lineup^39,40^. What is more, the morphing process protects the identity of the fillers which is legally required, for instance, in Germany: Photographs must be digitally manipulated so that the persons originally depicted in the photographs are no longer recognizable before these photographs may legally be used as filler photographs in lineups^41^. The downside of this practice is that it often produces morphing artifacts such as shadows, double edges, ghosting effects or blurring and lets the image appear softer^42,43^. In morphed lineups, the photograph of the suspect might therefore stand out from the fillers because it is the only photograph in the lineup that has not been digitally manipulated. Witnesses could thus use the absence of morphing artifacts as the cue to the identity of the suspect which might lead to a biased selection of the suspect.

In the present series of experiments, we examined the effect of the morphing manipulation in the mock-witness and eyewitness tasks. Whether morphed lineups are unfair was tested in Experiment 1 using the traditional mock-witness task, thereby relying on two classical measures of lineup fairness based on mock-witness choices, Tredoux’s *E* and the proportion of suspect selections. To anticipate, the results of the mock-witness task indicate that morphed simultaneous lineups are more unfair than non-morphed simultaneous lineups. In Experiments 2 to 4, we examined the effect of the morphing manipulation on eyewitness identification decisions using the 2-HT eyewitness identification model to measure biased suspect selection. In Experiment 2, we began by adding to the eyewitness task two features that are typical of the mock-witness task but highly unusual for the eyewitness task with the result that this version of the eyewitness task closely resembled the mock-witness task. These two features were then removed successively in Experiments 3 and 4 with the goal to identify the factors that may underlie the differences in the conclusions drawn based on data from the mock-witness task and the eyewitness task. Specifically, in Experiment 2, it was tested whether the biased-suspect-selection parameter *b* of the 2-HT eyewitness identification model reflects the unfairness of morphed lineups when participants (1) are discouraged from rejecting the lineups and (2) are alerted that a photograph might stand out from the other photographs in the lineup. When the eyewitness task thus closely resembled the mock-witness task, the eyewitness task led to the same conclusions as the mock-witness task: Biased suspect selection was enhanced in morphed simultaneous lineups in comparison to non-morphed simultaneous lineups. In the subsequent experiments, the procedure was brought closer to the standard procedure of typical eyewitness tasks. In Experiment 3, we removed the discouragement of lineup rejections. In Experiment 4, we removed both the discouragement of lineup rejections and the instruction to look for the photograph that stands out from the rest of the photographs. To anticipate, the results indicate that those who criticized the validity of the mock-witness task [e.g.,^21^] are correct: When the procedure was brought closer to the standard procedure of the eyewitness task, the effects of the morphing manipulation on biased suspect selection vanished. Specifically, the effect of the morphing manipulation on biased suspect selection was only descriptively present but not statistically significant in Experiment 3 and completely absent in Experiment 4. The results thus suggest that the mock-witness task has limited validity for drawing conclusions about eyewitness identification decisions. Instead, it is preferable to derive conclusions about lineup fairness directly from eyewitness identification decisions.

## Experiment 1

In comparison to the eyewitness task, the mock-witness task provides an impoverished data structure because mock witnesses are hindered from rejecting the lineup and have actually not seen the culprit so that mock-witness lineups are essentially culprit-absent lineups. With only two of the six data categories of the eyewitness task left, it is not possible to use the 2-HT eyewitness identification model introduced above to analyze the data of the mock-witness task. Therefore, we relied on traditional mock-witness measures—Tredoux’s *E* and the proportion of suspect selections—to measure the fairness of morphed and non-morphed simultaneous lineups in Experiment 1. However, in Experiment 2, the 2-HT eyewitness identification model was used to measure biased suspect selection in an eyewitness task that was modified to resemble the mock-witness task. To anticipate, the results obtained in the mock-witness task in Experiment 1 and the model-based analysis of eyewitness identification decisions in Experiment 2 converged in showing that morphed simultaneous lineups were significantly more unfair than non-morphed simultaneous lineups.

### Method

All experiments reported here were conducted online. They were implemented using *SoSci Survey*^44^ and were made available via https://www.soscisurvey.de. Participation was possible with a laptop or desktop computer, but not with a smartphone. All participants were recruited from the online research panel of Gapfish, Berlin, Germany (https://gapfish.com). Participants received a small monetary compensation for their participation.

#### Participants

Of the 851 participants who completed the socio-demographic questionnaire at the beginning of the experiment, 98 participants had to be excluded from the analysis because they did not complete the experiment or withdrew their consent to use their data (*n* = 91) or saw the lineups more than once due to repeated participation (*n* = 7). The final data set contained data from 753 participants (367 female, 384 male, 2 diverse) aged between 18 and 69 years (*M* = 45, *SD* = 14). The sample was characterized by a diversified level of education. We had aimed for a sample size of at least 750 valid datasets and ended data collection at the end of the day on which this criterion was met. Participants were randomly assigned to either the morphed lineup condition (*n* = 385) or the non-morphed lineup condition (*n* = 368).

#### Ethics statement

In each study, informed consent was obtained from all participants prior to participation. Ethical approval was received from the ethics committee of the Faculty of Mathematics and Natural Sciences at Heinrich Heine University Düsseldorf for a series of experiments of which the present experiments are a subset. All reported studies were carried out in accordance with the Declaration of Helsinki. In Experiments 2, 3 and 4, participants were warned that they would see a short video that included verbal and physical abuse. They were asked not to proceed if they felt uncomfortable expecting to watch such a video. At the end of the experiments, participants were informed that the crime they had witnessed had been staged.

#### Materials and procedure

Participants were told that a surveillance camera had recorded a crime scene in which four hooligans of a soccer club, FC Bayern München, attacked a soccer fan of a rivaling soccer club, Borussia Dortmund. Participants were informed that the police had constructed four lineups to test whether or not the suspects were the actual culprits. Participants received the instruction: “Each lineup consists of six pictures, one recent photo of a suspect and five photos from face databases” (all quotations in this article are translations of text that was originally presented in German). Participants were asked to indicate which lineup member was most likely to be the suspect in each lineup to help evaluate the fairness of the lineups. The instructions read: “We want to verify that the suspect’s photograph does not stand out from the other lineup members. If the photograph stands out, then you can recognize the suspect even if you are a person who had not seen the recording. Therefore, please look at all photographs carefully. Please select the person that you think is the suspect by clicking on the ‘Yes, is suspected’ button that belongs to the particular face”.

**Figure 2:**
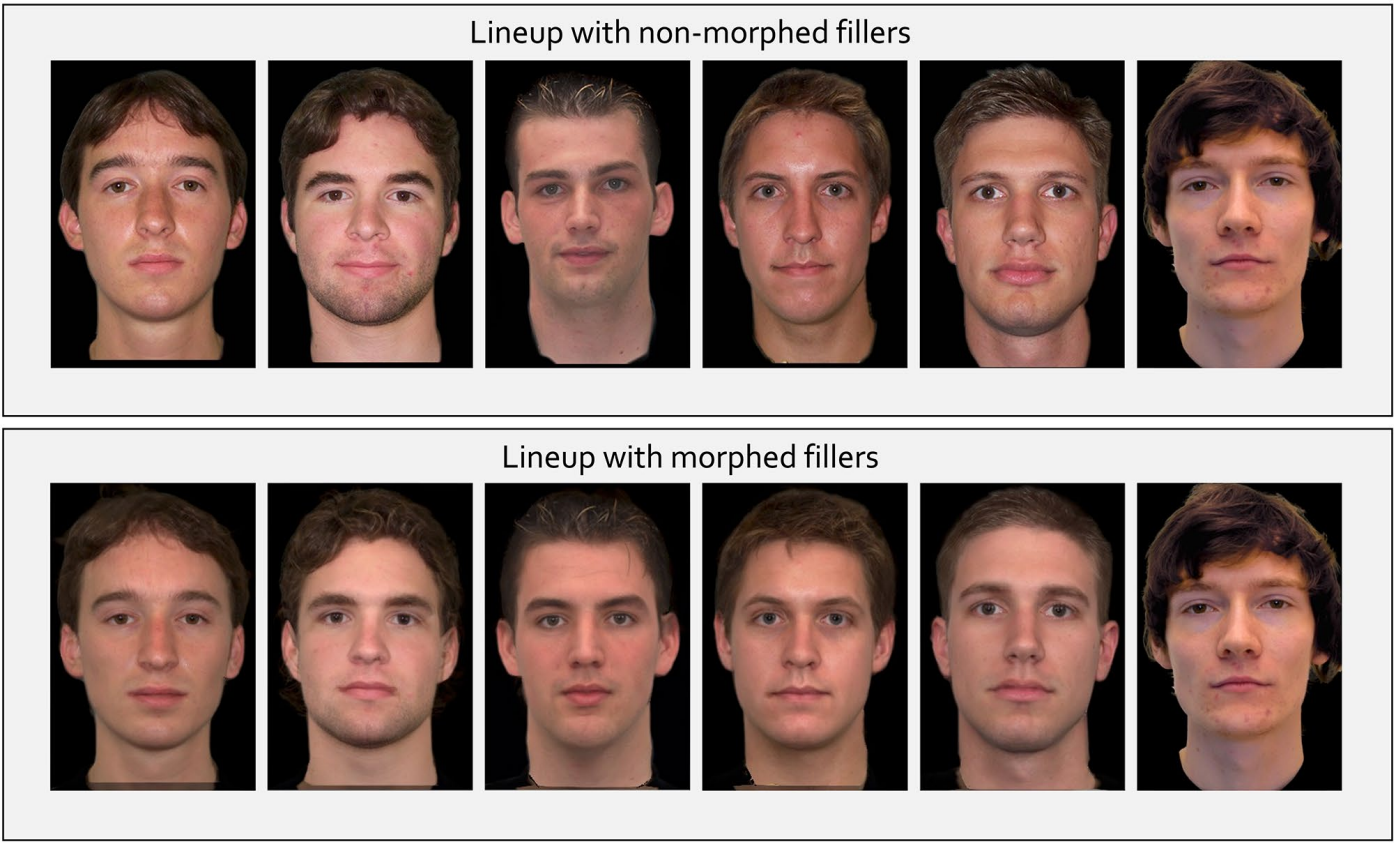
An illustration of a lineup with non-morphed and morphed fillers. The rightmost person represents the suspect but note that suspect and filler positions were always randomized in the experiments proper. We have written consent of the person representing the suspect to show the footage generated for the experiments. The photos of the fillers were taken from the Center for Vital Longevity Face Database^45^ and the Radboud Faces Database^46^, which are freely available for academic researchers.

Participants subsequently saw four separate lineups, each consisting of one suspect and five morphed or non-morphed fillers (for an example, see Fig. 2). In total, eight male white students were used as suspects who also served as culprits or innocent suspects in Experiments 2 to 4. The set of eight suspects consisted of four pairs of suspects who resembled each other in terms of basic physical characteristics (e.g., hair color, hairstyle, stature). For each lineup, one suspect from each pair of suspects was randomly selected to be presented in the lineup. This is parallel to how the lineups were constructed in Experiments 2 to 4.

**Figure 3:**
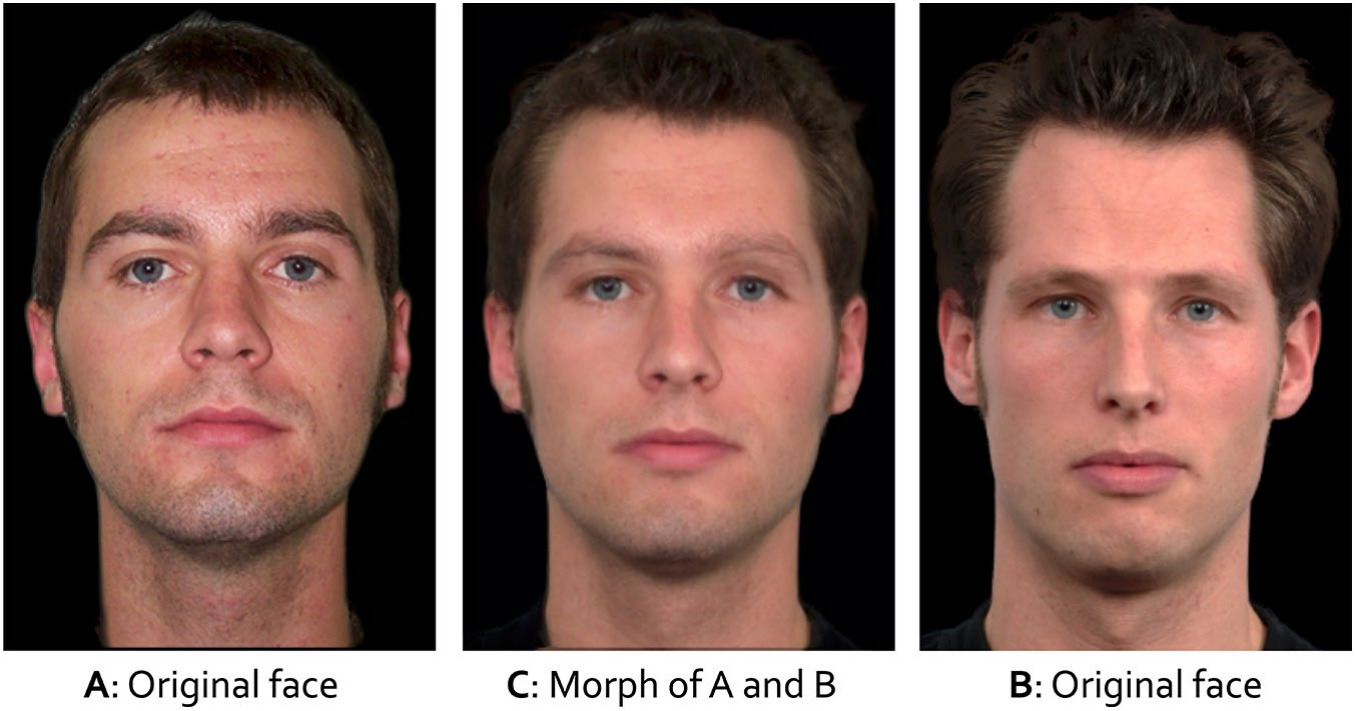
On the left side, an original filler face from Set A is shown. On the right side, an original filler face from Set B is shown. In the center, the face morph is shown (morph rate = 50:50). The photograph on the left was taken from the Center for Vital Longevity Face Database^45^. The photograph on the right was taken from the Radboud Faces Database^46^.

For the non-morphed lineup condition, five white male filler faces of persons aged between 18 and 29 years (hereinafter Set A) were chosen from the Center for Vital Longevity Face Database^45^ for each pair of suspects. To create the fillers for the morphed lineup condition, five additional white male filler faces of similar age (hereinafter Set B) were selected for each suspect pair. These faces were obtained from three face databases: The Center for Vital Longevity Face Database [^45^, https://agingmind.utdallas.edu/download-stimuli/face-database/], the FEI Face Database [^47^, https://fei.edu.br/~cet/facedatabase.html] and the Radboud Faces Database [^46^, http://www.rafd.nl]. All fillers were selected based on their similarity (as determined by the authors) to the corresponding suspects in terms of hair color, hairstyle and stature as well as their suitability for morphing (e.g., no glasses or piercings). Using *MorphAge* (Version 5.1, Creaceed, at https://creaceed.com/morphage), each filler from Set A was morphed with one filler from Set B by marking landmarks on one face (nose, eyes, eyebrows, mouth, ears, hairline and jaw-line) and matching each landmark to the corresponding point on the other face. Both faces of fillers from Set A and Set B were blended in a 50:50 ratio (i.e., a morph consisted of 50 % of each face). This procedure generated five morphed fillers for each suspect pair (for an example, see Fig. 3). All faces (i.e., those of the suspects and those of the fillers) were shown in frontal view against a black background with no clothes visible. All faces had a neutral facial expression. All photographs were edited to equate brightness, lighting and the position of the face among the photographs of the fillers and those of the suspects. The photographs were displayed at a resolution of 142 × 214 pixels.

The four lineups were presented one after another in a simultaneous format. In each lineup, all six faces were shown together in a single row with the option to respond “Yes, is suspected” appearing underneath each photograph. The position of the suspect and the five fillers was randomized. Implementing the typical mock-witness task^9^, participants were not given the option to reject the lineup. Once the participants had selected a person, they could proceed to the next lineup by pressing the “Next” button. The order in which the lineups appeared was randomly determined for each participant. After completing the four lineup trials, participants were debriefed and thanked for their participation. The experiment took about 10 min.

### Results

For each lineup, the distribution of mock-witness choices across the six lineup members was determined. Based on these mock-witness data, lineup fairness was computed in two ways. First, effective lineup size was assessed using Tredoux’s *E*, which provides an estimate of the number of plausible lineup members^19^. Tredoux’s *E* takes on a minimum value of 1 and a maximum value of *k,* the number of lineup members (in our lineups, six). Each lineup member who receives fewer choices than expected by chance will cause a reduction of the value of Tredoux’s *E,* starting from *k* and approaching 1. Tredoux’s *E* was calculated separately for each of the four morphed and non-morphed lineups before an average effective size was computed separately for the morphed and the non-morphed lineup condition that is reported below (details on the data underlying these effective sizes are reported in the Open Science Framework repository at https://osf.io/zaybc/). Second, the average proportion of suspect selections was calculated for both morphed and non-morphed lineups as a measure of biased selection of the suspect^9^. This measure is straightforward to interpret: If the mock-witness choices are equally distributed across the lineup members (i.e., one-sixth of the choices fall on the suspect), a lineup would be considered perfectly fair. If a disproportionate number of mock witnesses pick out the suspect, a lineup is considered unfair. Thus, a greater proportion of participants choosing the suspect from morphed lineups than from non-morphed lineups would indicate that the morphed lineups are more biased toward the suspect than the non-morphed lineups.

The average Tredoux’s *E* was higher for the non-morphed lineup condition (*M* = 4.51) than for the morphed lineup condition (*M* = 3.44), indicating that the morphed lineups were more unfair than the non-morphed lineups. The same conclusion can be reached when calculating the proportion of suspect selections in both conditions. The average proportion of suspect selections was significantly higher in the morphed lineup condition (*M* = 47.5 %) than in the non-morphed lineup condition (*M* = 25 %), as determined by a *z*-test for proportions (*z* = 12.80, *p* < 0.001).

### Discussion

The results obtained in the traditional mock-witness task indicate that the morphed lineups were more unfair than the non-morphed lineups. These results thus lead to the conclusion that the police should stop using this morphing technique as it leads to artifacts that make the suspect stand out from the other lineup members. However, it has yet to be shown whether or not these findings are limited to the mock-witness task. Therefore, the purpose of Experiments 2 to 4 was to examine the effects of the same morphing manipulation on eyewitness identification decisions in simultaneous and sequential lineups.

## Experiment 2

It cannot be taken for granted that the mock-witness choices validly reflect the processes that determine eyewitness identification decisions. Therefore, it has to be tested whether the morphing manipulation affects eyewitness identification decisions to the same extent as it affects mock-witness choices. As noted above, the mock-witness task differs from a typical eyewitness task in at least two significant ways. Unlike eyewitnesses, mock witnesses (1) are required to choose one of the lineup members and (2) are alerted to the fact that one lineup member might stand out from the others. Therefore, the aim of the following series of experiments was to test, across experiments, whether evidence for the unfairness of the morphed lineups emerged depending on whether these two features were present in the eyewitness task.

As the next step, we aimed at testing whether the unfairness effects of the morphing manipulation could be demonstrated in eyewitness identification decisions when the eyewitness task was modified to mimic the mock-witness task—that is, when participants (1) were discouraged from rejecting the lineup and (2) were alerted to the fact that the suspect may stand out from the fillers. The eyewitness task provides a richer data structure than the mock-witness task because suspect identifications, filler identifications and lineup rejections in both culprit-present and culprit-absent lineups can be observed. It is thus important to rely on a measurement model that takes the full data structure of the eyewitness task into account. The 2-HT eyewitness identification model capitalizes on the full range of data categories that can be observed in the eyewitness task. It has been successfully demonstrated that the model’s parameter *b* sensitively reflects the biased selection of suspects^32,34^ and can thus be used to assess the unfairness of lineups. If the biased-suspect-selection parameter *b* is sensitive to the morphing manipulation used in Experiment 1, the estimate of parameter *b* should be higher for simultaneous morphed lineups than for simultaneous non-morphed lineups.

An additional aspect not mentioned so far is that the mock-witness technique has been proposed to evaluate the fairness of simultaneous lineups but it is of limited use in estimating the fairness of sequential lineups^21,48^. However, in some countries such as the UK and Germany, the sequential presentation has become the standard way of conducting police lineups^49,50^. The second aim of Experiment 2 was thus to test the effect of the morphing manipulation on biased suspect selection in sequential lineups. Here it is useful that the 2-HT model has been demonstrated to validly reflect biased selection in both simultaneous and sequential lineups^32^. Previous research has demonstrated that sequential lineups provide some protection against biased suspect selection in unfair lineups^3,51,52^. For example, in simultaneous lineups, a photograph that differs from the other photographs in brightness, contrast, color balance or softness may pop out from the others. In sequential lineups, witnesses cannot compare the photographs side-by-side. Therefore, it may be less salient that one photograph stands out from the others in sequential lineups. There is thus reason to expect that the morphing manipulation is less likely to affect eyewitness identification decisions in sequential lineups than in simultaneous lineups. As a consequence, biased selection of the suspect may only be enhanced in simultaneous morphed lineups in comparison to simultaneous non-morphed lineups but may not differ between morphed and non-morphed sequential lineups.

### Method

#### Participants

Of the 934 participants who completed the socio-demographic questionnaire at the beginning of the experiment, 151 participants had to be excluded from the analysis because they did not complete the experiment or withdrew their consent to use their data (*n* = 120), incorrectly answered the attention-check question (*n* = 11) or watched the video more than once due to repeated participation (*n* = 20). The final data set contained data from 783 participants (336 female, 445 male, 2 diverse) aged between 18 and 69 years (*M* = 45, *SD* = 14). The sample was characterized by a diversified level of education. We had aimed for a sample size of at least 750 valid datasets and stopped data collection at the end of the day on which this criterion was met. A sensitivity analysis using G*Power^53^ showed that with a sample size of *N* = 783, four eyewitness identification decisions and an alpha error probability of 0.05, it was possible to detect even small effects of the morphing manipulation on the biased-suspect-selection parameter *b* of effect size *w* = 0.06 with a statistical power (1 − beta error probability) of 0.95. Participants were randomly assigned to one of the four lineup conditions: the morphed simultaneous lineup condition (*n* = 199), the non-morphed simultaneous lineup condition (*n* = 190), the morphed sequential lineup condition (*n* = 196) and the non-morphed sequential lineup condition (*n* = 198).

#### Materials and procedure

##### Staged-crime videos

The same two parallel videos were used (henceforth referred to as Video A and Video B) as in the validation experiments of Winter et al.^32^. Both videos showed the same staged crime, but with different sets of actors: Four hooligans of the German soccer club FC Bayern München (henceforth referred to as the culprits) verbally and physically attacked a fan of the rival soccer club Borussia Dortmund (henceforth referred to as the victim) at a bus stop. The hooligans and their victim wore typical fan clothing of the soccer clubs (caps, shirts and scarfs in typical club colors). The four culprits poked fun at the victim, insulted him and tossed his personal belongings around. At the end of the video, the culprits pushed the victim to the ground. The four culprits continued to verbally and physically abuse the victim. Once the culprits noticed another pedestrian approaching (not visible in the videos), they ran away shouting loudly. Note that in many other lineup studies, participants are only exposed to a single culprit at encoding and thus to a single lineup at test [e.g.,^8,54–56^]. However, to increase the efficiency of data collection, we followed the lead of other researchers [e.g.,^57–60^] and presented our participants with a video showing four culprits. This procedure allowed us to generate four data points per participant instead of just one. Also note that multiple-culprit crimes are in fact quite frequent^61,62^. For instance, in 2008, 25 % of all crimes committed in the UK involved four or more culprits [^63^, p. 287].

The two parallel videos had the same content (i.e., the videos contained the same verbal and physical abuse in the same sequence and with the same timing), but the culprits and the victims differed between the two videos. Care was taken to select the actors in such a way that the victim of Video A resembled the victim of Video B as closely as possible and that each of the four culprits of Video A resembled one of the four culprits of Video B in hair color, hairstyle and stature (i.e., Culprit 1 in Video A matched Culprit 1 in Video B, Culprit 2 in Video A matched Culprit 2 in Video B and so on). Note that the same eight faces had served as suspects in Experiment 1 to ensure comparability between the experiments. It was randomly determined whether participants watched Video A or Video B. Both videos were 130 s long and showed a clear view of the culprits’ faces. The videos were presented in a resolution of 885 × 500 pixels.

Participants could start the video by clicking on the “Start” button. They were unable to proceed to the next page until they had watched the whole video. After the video had finished, participants had to answer a 10-alternatives attention-check question (“What kind of people were shown in the video?”; correct response: “Soccer fans”) to test whether participants had been paying attention to the video. The order of the response options was randomized.

##### Lineup procedures

Participants who had correctly answered the attention-check question were asked to identify the four culprits: “The video you just watched showed aggressive FC Bayern München hooligans. You will be asked to identify these hooligans. For this purpose, we are going to show you some lineups”*.* As in Experiment 1, participants were informed that “Each lineup consists of six pictures, one recent photo of the suspect and five photos taken from face databases”. They were also made aware of the possibility that the suspect might stand out from the other lineup members: “It is possible that the suspect stands out from the other lineup members. If the suspect stands out, then you can recognize the suspect even if you have not seen the video. Therefore, please look at all photos carefully”. Lineup rejections were discouraged by instructing the participants: “It is very likely that every lineup contains a culprit. Therefore, even if you are uncertain about whether or not the culprit is in the lineup, choose the picture that stands out among the others. Then you will almost certainly identify the culprit. To do this, click on the ‘Yes, was present’ button that belongs to that face. Only if you are very certain that the persons do [simultaneous lineups]/person does [sequential lineups] not represent any of the culprits, click on the ‘No, none of these persons was present’ [simultaneous lineups]/‘No, this person was not present’ [sequential lineups] button”*.* Participants were not made aware of the number of lineups that were about to follow.

Participants saw a total of four separate lineups, two were randomly selected to be culprit-present lineups and the other two were culprit-absent lineups. The lineups consisted of the same faces as in Experiment 1. Each lineup consisted of the facial photographs of six persons, one suspect face and either five morphed or five non-morphed filler faces (see Fig. 2). The crossed lineup procedure introduced by Winter et al.^32^ was used to manipulate the suspect’s guilt. Specifically, if participants had seen Video A, two culprits of Video A (e.g., Culprits 1 and 3) served as the culprits in the culprit-present lineups, while two culprits of Video B (e.g., Culprits 2 and 4) served as the innocent suspects in the culprit-absent lineups. If participants had seen Video B, two culprits of Video B served as the culprits in the culprit-present lineups while two culprits of Video A served as the innocent suspects in the culprit-absent lineups. This approach had two advantages: First, culprit-absent lineups contained a designated innocent suspect to whom the fillers had been matched. This situation represents a more ecologically valid lineup procedure than using only fillers in culprit-absent lineups. This is so because, in practice, the photographs of the suspects (whose guilt or innocence is unknown) are taken from other sources (e.g., social media) than the photographs of the fillers which are usually taken from face databases and may be digitally altered. Second, culprit-present and culprit-absent lineups included the identical filler faces; only the identity of the suspect was changed. Which of the two suspects served as the culprit or innocent suspect depended on the random assignment to one of the two videos (see above). In that way, it was ensured that, on average, the degree of fairness was the same in culprit-present and culprit-absent lineups. A similar approach, the single-lineup procedure, has been proposed by Oriet and Fitzgerald^64^. In contrast to the crossed lineup procedure used here, the single lineup procedure implies showing all participants the same lineup after having seen one of two videos, one that contains the suspect in the lineup while the other contains a person who is not presented in the lineup but matches the physical description of the suspect. As in Experiment 1, all photographs were presented at a resolution of 142 × 214 pixels.

Participants were randomly assigned to either the simultaneous or the sequential lineup conditions. In the simultaneous lineup conditions, the six faces were shown together in one row. Participants made a decision by either clicking on the “Yes, was present” button underneath a face to identify a person as a culprit or by clicking on the “No, none of these persons was present” button located to the right of each lineup to reject the lineup. After having made a decision, participants were asked to express how confident they were in their judgments in order to approximate the procedure to that of a real police lineup. Then they could initiate the presentation of the next lineup by clicking on the “Next” button. In the sequential lineup conditions, the faces were presented one at a time. For each of the six faces, participants decided whether or not the depicted face belonged to one of the culprits by clicking on either the “Yes, was present” button underneath the face or the “No, this person was not present” button located to the right of the face. A decision was required before participants could proceed to the next lineup member. If participants identified more than one face within a single lineup, only the last identification decision was counted. This procedure is legally prescribed in several jurisdictions such as Germany or the United States^24,50,65^. It also corresponds to the identification procedure in the simultaneous lineups in which it was possible for participants to revise their decision before clicking the “Next” button. After each decision, participants were asked to indicate their level of confidence in their judgment in the same manner as in the simultaneous lineup conditions. A lineup was counted as rejected if participants identified none of the lineup members. The order with which the lineups were presented was randomized, as was the position of the lineup members in each lineup. After their response to the fourth lineup, participants were debriefed and thanked. The experiment took about 10 min.

### Results

Four instances of the model illustrated in Fig. 1 were used for the model-based analysis, one for the simultaneous morphed lineups, one for the simultaneous non-morphed lineups, one for the sequential morphed lineups and one for the sequential non-morphed lineups. Goodness-of-fit tests and parameter estimates were calculated using *multiTree*^66^. The alpha error probability was set to 0.05. The observed response frequencies and proportions for Experiments 2, 3 and 4 are reported in Table 1. Our goal was to start with a base model that was as simple as possible. Therefore, we imposed restrictions onto the 2-HT eyewitness identification model that seemed justified, if not required on a priori grounds, to achieve this goal. First, there was no a priori reason why the ability to detect the absence of the culprit should differ as a function of the conditions in the present experiment [see^32,34^ for conditions that can be expected to affect the probability of culprit-absence detection]. Therefore, the parameter representing culprit-absence detection (*dA*) was set to be equal across the four lineup conditions. Second, there was no a priori reason why the ability to detect the presence of the culprit should differ between the morphed and non-morphed lineups. However, previous results that were obtained with the same stimulus materials and procedure^32^ suggest that culprit-presence detection is somewhat better in simultaneous than in sequential lineups. Therefore, the culprit-presence-detection parameter *dP* was set to be equal between the simultaneous morphed and non-morphed conditions and between the sequential morphed and non-morphed conditions. Third, there was no a priori reason why guessing-based selection should differ between the morphed and non-morphed lineups. However, previous results^32^ suggest that guessing-based selection is enhanced in sequential in comparison to simultaneous lineups. Therefore, the guessing-based-selection parameter *g* was set to be equal between the simultaneous morphed and non-morphed conditions and between the sequential morphed and non-morphed conditions. The asymptotically chi-square distributed likelihood-ratio goodness-of-fit statistic (with degrees of freedom reported in parentheses) [for details, see^67^] indicated that the base model incorporating these restrictions fit the data, *G*^2^(7) = 2.55, *p* = 0.924.

**Table 1:** Observed response frequencies and proportions (in parentheses) as a function of lineup format and the type of lineup fillers observed in Experiments 2, 3 and 4.

Lineup format	Type of lineup fillers	Culprit-present lineups			Culprit-absent lineups		
		Culprit identifications	Filler identifications	Lineup rejections	Innocent- suspect identifications	Filler identifications	Lineup rejections
Experiment 2							
Simultaneous	Morphed	177 (0.44)	120 (0.30)	101 (0.25)	93 (0.23)	161 (0.40)	144 (0.36)
	Non-morphed	155 (0.41)	117 (0.31)	108 (0.28)	66 (0.17)	160 (0.42)	154 (0.41)
Sequential	Morphed	112 (0.29)	220 (0.56)	60 (0.15)	58 (0.15)	247 (0.63)	87 (0.22)
	Non-morphed	130 (0.33)	201 (0.51)	65 (0.16)	67 (0.17)	234 (0.59)	95 (0.24)
Experiment 3							
Simultaneous	Morphed	154 (0.39)	117 (0.30)	123 (0.31)	70 (0.18)	131 (0.33)	193 (0.49)
	Non-morphed	132 (0.37)	95 (0.26)	133 (0.37)	47 (0.13)	135 (0.38)	178 (0.49)
Sequential	Morphed	116 (0.30)	208 (0.53)	66 (0.17)	53 (0.14)	235 (0.60)	102 (0.26)
	Non-morphed	126 (0.34)	184 (0.49)	66 (0.18)	58 (0.15)	220 (0.59)	98 (0.26)
Experiment 4							
Simultaneous	Morphed	135 (0.37)	86 (0.23)	145 (0.40)	49 (0.13)	119 (0.33)	198 (0.54)
	Non-morphed	144 (0.36)	119 (0.30)	137 (0.34)	55 (0.14)	139 (0.35)	206 (0.52)
Sequential	Morphed	125 (0.32)	189 (0.49)	74 (0.19)	69 (0.18)	209 (0.54)	110 (0.28)
	Non-morphed	126 (0.34)	163 (0.44)	79 (0.21)	62 (0.17)	199 (0.54)	107 (0.29)

The proportions are rounded to two decimal places and therefore do not always add up exactly to 1.

In an MPT model such as the 2-HT eyewitness identification model, hypotheses can be tested directly at the level of the parameters representing the postulated processes. For instance, the hypothesis that biased suspect selection is higher in the morphed lineup conditions than in the non-morphed lineup conditions can be tested by restricting parameter *b* to be equal between these conditions. If the model with this restriction fits significantly worse to the data than the base model (measured by the ∆*G*^2^ difference statistic with degrees of freedom corresponding to the difference between degrees of freedom of the model with the additional restriction and the degrees of freedom of the base model), we would have to reject the equality assumption implied by the restriction and would conclude that parameter* b* differs between conditions.

**Figure 4:**
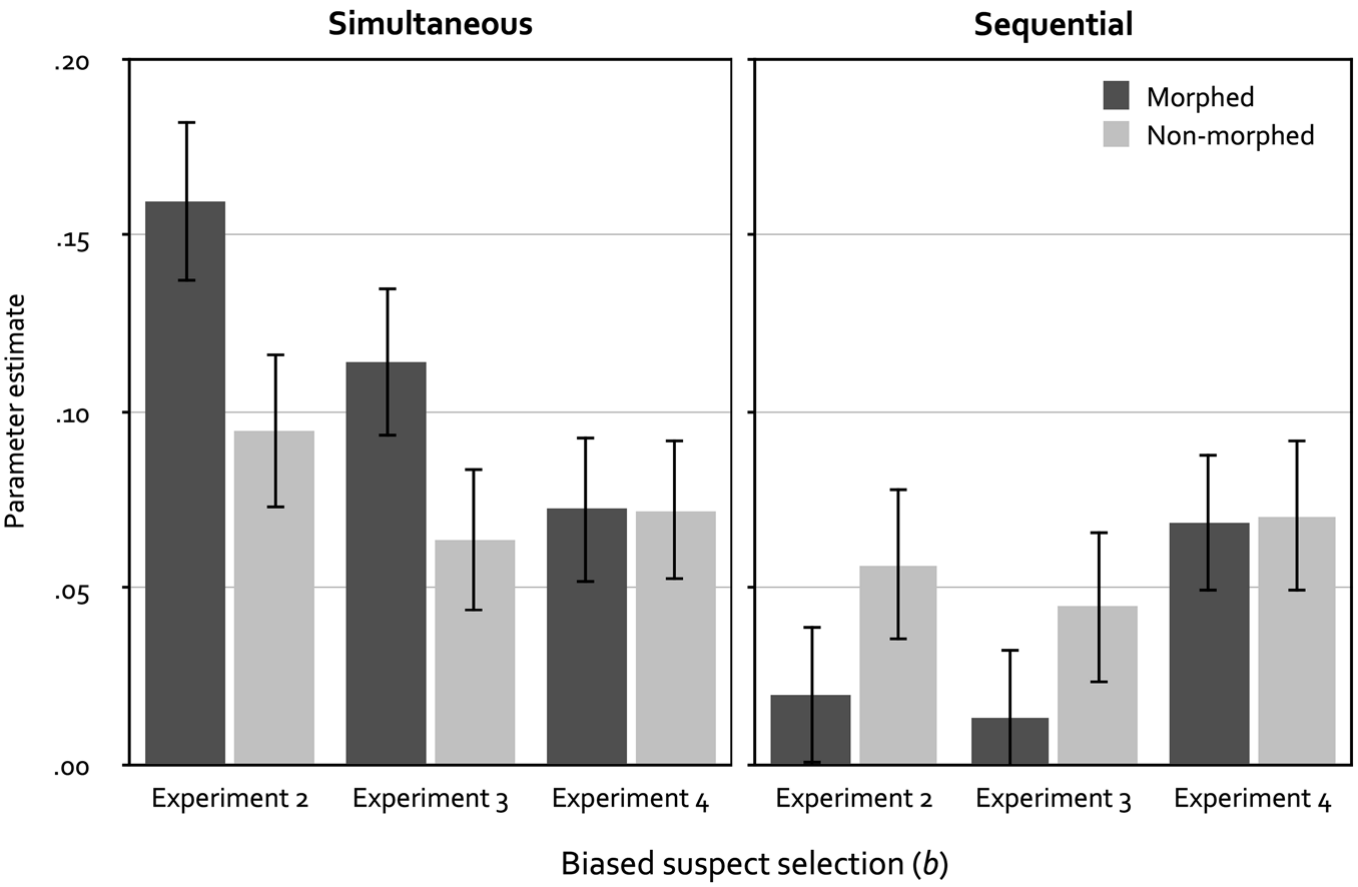
Parameter estimates of parameter *b* reflecting the probability of biased suspect selection as a function of lineup format (simultaneous vs. sequential) and the type of lineup fillers (morphed vs. non-morphed) in Experiments 2, 3 and 4. The error bars represent the standard errors.

Figure 4 shows the parameter estimates of the biased-suspect-selection parameter *b* for morphed and non-morphed lineups as a function of lineup format. The probability of biased suspect selection was higher for morphed lineups than for non-morphed lineups when simultaneous lineups were used, ∆*G*^2^(1) = 5.31, *p* = 0.021, *w* = 0.04, in accordance with the mock-witness results of Experiment 1. However, biased suspect selection did not differ between morphed and non-morphed lineups when sequential lineups were used, ∆*G*^2^(1) = 2.04, *p* = 0.153, *w* = 0.03. In addition, the probability of biased suspect selection was significantly higher for morphed simultaneous than for morphed sequential lineups, ∆*G*^2^(1) = 21.89, *p* < 0.001, *w* = 0.08, but it did not differ between non-morphed simultaneous and sequential lineups, ∆*G*^2^(1) = 1.60, *p* = 0.207, *w* = 0.02.

The parameter estimates for culprit-presence detection (*dP*), guessing-based selection (*g*) and culprit-absence detection (*dA*) as a function of lineup format are shown in Table 2. Given that our hypotheses pertained only to biased suspect selection (*b*), we do not include an analysis or discussion of the other parameters here. However, we will provide a brief overview and interpretation of the results of Experiments 2 to 4 pertaining to culprit-presence detection and guessing-based selection in the General Discussion.

**Table 2:** Parameter estimates for culprit-presence detection (*dP*), guessing-based selection (*g*) and culprit-absence detection (*dA*) as a function of lineup format (Experiments 2, 3 and 4).

Lineup format	Experiment 2			Experiment 3			Experiment 4		
	*dP*	*g*	*dA*	*dP*	*g*	*dA*	*dP*	*g*	*dA*
Simultaneous	0.27 (0.03)	0.59 (0.02)	0.04 (0.02)	0.26 (0.02)	0.50 (0.02)	0.06 (0.03)	0.26 (0.02)	0.46 (0.02)	0.06 (0.03)
Sequential	0.17 (0.02)	0.80 (0.02)		0.19 (0.02)	0.78 (0.02)		0.18 (0.03)	0.74 (0.02)	

Within the base model, parameters *dP* and *g* were each set to be equal between morphed and non-morphed lineups, separately for simultaneous and sequential lineups. Parameter *dA* was set to be equal among the four lineup conditions. Values in parentheses represent the standard errors. See text for details.

### Discussion

In Experiment 2, it was tested whether the morphing manipulation that affected mock-witness-based measures of unfairness in Experiment 1 would affect the biased selection of the suspect that was directly estimated from the identification decisions of eyewitnesses in simultaneous and sequential lineups if the eyewitness task closely resembled the mock-witness task. Eyewitnesses were discouraged from rejecting the lineup and were made aware of the fact that the suspect may stand out from the fillers. Under these conditions, the model-based results converged with those based on the mock-witness measures obtained in Experiment 1. In simultaneous lineups, the morphing manipulation significantly increased the biased selection of the suspect, represented by parameter *b* of the 2-HT eyewitness identification model.

By using the 2-HT eyewitness identification model it was also possible to measure the effect of the morphing manipulation on biased suspect selection in sequential lineups. The probability of biased suspect selection did not differ significantly between morphed and non-morphed lineups when they were presented sequentially. In sequential lineups, it is not possible to compare the photographs side by side; participants can only look at each lineup member individually. Without the direct comparison of all of the photographs in the lineup, it may have been difficult or even impossible for the participants to identify the fact that it is the absence of morphing artifacts that distinguishes the suspect from the fillers. In consequence, biased suspect selection was less prevalent in sequential lineups than in simultaneous lineups. This is in line with previous research indicating that sequential lineups provide some protection against biased suspect selection in unfair lineups^3,51,52^, presumably because participants cannot compare lineup faces side-by-side and thus are unable to detect the cues that distinguish the suspect from the fillers.

In Experiment 2, eyewitnesses were used instead of mock witnesses, but we discouraged participants from rejecting the lineup and alerted them to the fact that one lineup member might stand out from the others. This is typical for instructions that are used in the mock-witness task but deviates from the pre-lineup instructions that are recommended for the eyewitness task^26–28^. Experiments 3 and 4 serve to test whether the same or different results are obtained when the procedure is brought closer to the standard procedure of typical eyewitness tasks.

## Experiment 3

Experiment 3 was identical to Experiment 2 with the exception that the pre-lineup instructions did not discourage participants from rejecting the lineup. Previous research has consistently demonstrated that one-sided instructions that emphasize only the need to identify the culprit but ignore the need to reject culprit-absent lineups enhance both correct culprit identifications and false innocent-suspect identifications^29,68^. The requirement to identify one of the lineup members as the culprit may increase the likelihood that participants make the non-memory-based identification decision to identify the person who stands out from the other persons in the lineup. It is unclear whether participants rely on the morphing artifacts when they receive two-sided instructions that emphasize both the need to identify the culprit and the need to reject culprit-absent lineups. It seemed thus interesting to test whether biased suspect selection is enhanced in simultaneous morphed lineups in comparison to simultaneous non-morphed lineups in Experiment 3 in which participants were alerted to the fact that the face of the suspect might stand out from the other faces but participants were not discouraged from rejecting the lineup.

### Method

#### Participants

Of the 894 participants who completed the socio-demographic questionnaire at the beginning of the experiment, 134 had to be excluded from the analysis because they did not complete the experiment or withdrew their consent to use their data (*n* = 112), incorrectly answered the attention-check question (*n* = 13) or watched the video more than once due to repeated participation (*n* = 9). The final data set contained data from 760 participants (324 female, 434 male, 2 diverse) aged between 18 and 85 years (*M* = 45, *SD* = 15). The sample was characterized by a diversified level of education. We had aimed for a sample size of at least 750 valid datasets and stopped data collection at the end of the day on which this criterion was met. A sensitivity analysis showed that with a sample size of *N* = 760 participants, four eyewitness identification decisions and an alpha error probability of 0.05, it was possible to detect even small effects of the morphing manipulation on the biased-suspect-selection parameter *b* of effect size *w* = 0.07 with a statistical power (1 − beta error probability) of 0.95^53^. As in Experiment 2, participants were randomly assigned to one of the four lineup conditions: the morphed simultaneous lineup condition (*n* = 197), the non-morphed simultaneous lineup condition (*n* = 180), the morphed sequential lineup condition (*n* = 195) and the non-morphed sequential lineup condition (*n* = 188).

#### Materials and procedure

Materials and procedure were identical to those of Experiment 2 with the following exception. Instead of discouraging lineup rejections by implying that the culprit would be present in the lineup, the instructions emphasized the possibility that the culprit might not be present. As in Experiment 2, however, participants were alerted that a photograph might stand out from the other photographs in the lineup. The instructions read: “The video you just watched showed aggressive FC Bayern München hooligans. You will be asked to identify these hooligans. For this purpose, we are going to show you some lineups. Each lineup consists of six pictures, one recent photo of the suspect and five photos taken from face databases. It is possible that the suspect stands out from the other lineup members. If the suspect stands out, then you can recognize the suspect even if you have not seen the video. Therefore, please look at all photos carefully. You will be asked to indicate if one of the lineup members is one of the FC Bayern München hooligans shown in the video. It is also possible that none of the hooligans is in the lineup. If you recognize a face, then click on the ‘Yes, was present’ button that belongs to that face. Otherwise click on the ‘No, none of these persons was present’ [simultaneous lineups]/‘No, this person was not present’ [sequential lineups] button”.

### Results

The same assumptions as in Experiment 2 were used to arrive at the base model. This base model fit the data, *G*^2^(7) = 3.58, *p* = 0.827.

Figure 4 shows the parameter estimates of the biased-suspect-selection parameter *b* for morphed and non-morphed lineups as a function of lineup format. Parallel to Experiment 2, the probability of biased suspect selection was descriptively higher for morphed simultaneous lineups compared to non-morphed simultaneous lineups, but in contrast to Experiment 2 this difference was no longer statistically significant, ∆*G*^2^(1) = 3.63, *p* = 0.057, *w* = 0.03. As in Experiment 2, biased suspect selection did not differ between morphed and non-morphed lineups when sequential lineups were used, ∆*G*^2^(1) = 1.49, *p* = 0.222, *w* = 0.02. Also as in Experiment 2, the probability of biased suspect selection was significantly higher for morphed simultaneous than for morphed sequential lineups, ∆*G*^2^(1) = 12.53, *p* < 0.001, *w* = 0.06, but it did not differ between non-morphed simultaneous and sequential lineups, ∆*G*^2^(1) = 0.42, *p* = 0.515, *w* = 0.01.

The parameter estimates for culprit-presence detection (*dP*), guessing-based selection (*g*) and culprit-absence detection (*dA*) as a function of lineup format are shown in Table 2.

### Discussion

In Experiment 3, the probability of biased suspect selection no longer differed significantly between morphed and non-morphed simultaneous lineups. Emphasizing that the culprit might or might not be in the lineup reduced the probability of biased suspect selection in simultaneous morphed lineups compared to Experiment 2 in which lineup rejections were discouraged. This was expected given the plausible assumption that instructions discouraging lineup rejections cause eyewitnesses to search harder than they usually do for cues that make the suspect stand out.

However, at a descriptive level the probability of biased suspect selection was still larger in morphed simultaneous lineups compared to non-morphed simultaneous lineups. Moreover, when the fillers were morphed, biased suspect selection was still less prevalent in sequential lineups than in simultaneous lineups, which is consistent with the results of Experiment 2 as well as previous research^3,51,52^. Thus, there was still some evidence of an effect of the morphing manipulation on the data obtained in Experiment 3.

## Experiment 4

Experiment 4 was identical to Experiment 3 with the exception that participants were not alerted to the fact that the suspect’s photograph might stand out from the other photographs in the lineup. Instead, participants were presented with instructions that are given in a typical eyewitness task in which participants are not discouraged from rejecting the lineup and are not alerted that a photograph might stand out from the other photographs in the lineup^26–28^. The main question was whether the effect of the morphing manipulation on biased suspect selection in simultaneous lineups would be abolished under these conditions that, within the present series of experiments, most closely mirror real police lineup procedures.

### Method

#### Participants

Of the 958 participants who completed the socio-demographic questionnaire at the beginning of the experiment, 197 participants had to be excluded from the analysis because they did not complete the experiment or withdrew their consent to use their data (*n* = 155), incorrectly answered the attention-check question (*n* = 13), watched the video more than once due to repeated participation (*n* = 22) or a technical error occurred during the experiment (*n* = 7). The final data set contained data from 761 participants (335 female, 426 male) aged between 18 to 80 years (*M* = 48, *SD* = 17). The sample was characterized by a diversified level of education. We had aimed for a sample size of at least 750 participants and stopped data collection at the end of the day on which this criterion was met. A sensitivity analysis showed that with a sample size of *N* = 761 participants, four eyewitness identification decisions and an alpha error probability of 0.05, it was possible to detect even small effects of the morphing manipulation on the biased-suspect-selection parameter *b* of effect size *w* = 0.07 with a statistical power (1 − beta error probability) of 0.95^53^. Participants were randomly assigned to one of the four lineup conditions: the morphed simultaneous lineup condition (*n* = 183), the non-morphed simultaneous lineup condition (*n* = 200), the morphed sequential lineup condition (*n* = 194) and the non-morphed sequential lineup condition (*n* = 184).

#### Materials and procedure

Materials and procedure were identical to those of Experiment 3 with the exception that the instructions no longer alerted participants that a photograph might stand out from the other photographs in the lineup and thus corresponded to those used in typical eyewitness identification situations. The instructions read: “The video you just watched showed aggressive FC Bayern München hooligans. You will be asked to identify these hooligans. For this purpose, we are going to show you some lineups. In each lineup, you will see some faces. You will be asked to indicate if one of the lineup members is one of the FC Bayern München hooligans shown in the video. It is also possible that none of the hooligans is in the lineup. If you recognize a face, then click on the ‘Yes, was present’ button that belongs to that face. Otherwise, click on the ‘No, none of these persons was present’ [simultaneous lineups]/‘No, this person was not present’ [sequential lineups] button”.

### Results

The same assumptions as in Experiments 2 and 3 were used to arrive at the base model. This base model fit the data, *G*^2^(7) = 6.61, *p* = 0.471.

Figure 4 shows the parameter estimates of the biased-suspect-selection parameter *b* for morphed and non-morphed lineups as a function of lineup format. Crucially, the descriptive difference between morphed and non-morphed simultaneous lineups that was still evident in Experiment 3 was absent in Experiment 4. This result is so clear from the sizes of the parameter estimates that it does not require a statistical test, but for completeness, we report here that the probability of biased suspect selection did not differ significantly between morphed simultaneous lineups and non-morphed simultaneous lineups, ∆*G*^2^(1) < 0.01, *p* = 0.992, *w* < 0.01. Further, as in Experiments 2 and 3, biased suspect selection did not differ between morphed and non-morphed lineups when sequential lineups were used, ∆*G*^2^(1) < 0.01, *p* = 0.950, *w* < 0.01. Finally, the probability of biased suspect selection differed neither between morphed simultaneous and sequential lineups, ∆*G*^2^(1) = 0.02, *p* = 0.898, *w* < 0.01, nor between non-morphed simultaneous and sequential lineups, ∆*G*^2^(1) < 0.01, *p* = 0.952, *w* < 0.01.

The parameter estimates for culprit-presence detection (*dP*), guessing-based selection (*g*) and culprit-absence detection (*dA*) as a function of lineup format are shown in Table 2.

### Discussion

In Experiment 4, the effect of the morphing manipulation on biased suspect selection was completely absent. When lineup rejections were not discouraged and participants were not alerted that a photograph might stand out from the other photographs in the lineup, the probability of biased suspect selection did not differ between morphed and non-morphed lineups in both simultaneous and sequential formats. In contrast to Experiments 2 and 3, the probability of biased suspect selection was comparable between simultaneous and sequential lineups even in the morphed lineup condition. Thus, when the task characteristics closely mirrored the conditions of a real lineup procedure, there was absolutely no evidence of an effect of the morphing manipulation on biased suspect selection in any of the lineups.

## General discussion

The well-validated 2-HT eyewitness identification model^32,34^ allows measuring lineup fairness directly from eyewitness identification decisions without relying on the choices of mock witnesses. The problem with using mock witnesses is that their task differs substantially from the task of eyewitnesses. As a consequence, there are doubts as to whether lineup fairness measured in the mock-witness task can predict lineup fairness in a typical eyewitness task^10,15,20,21,48^. The present series of experiments demonstrates that these doubts are justified.

We measured the fairness of lineups containing either morphed or non-morphed fillers. This morphing manipulation is of applied relevance considering that the morphing technique can serve as a method both to create fillers when the pertinent databases do not contain enough photographs that are similar enough to descriptions of the suspect^39,40^ and to morph photographs to protect the identities of the persons depicted in the filler photographs. The latter is required, for instance, in Germany^41^. These practical advantages notwithstanding, morphing also comes with potential disadvantages in that artifacts may arise during the morphing process^42,43^. Given that only the photographs of the fillers are digitally manipulated while the photograph of the suspect is not, the absence of morphing artifacts can serve as a cue to the identity of the suspect. In the worst case, these morphing artifacts could lead to unfair lineups from which witnesses may choose the suspect not because they recognize the suspect’s face but because the suspect’s facial photograph can be identified without relying on memory. We started by examining the fairness of morphed and non-morphed lineups using measures that were obtained from the traditional mock-witness task. From the mock-witness choices, we calculated Tredoux’s *E* and the proportion of suspect selections as the most prominent measures of effective lineup size and biased suspect selection, respectively. Both measures provided evidence that morphed lineups were more unfair than non-morphed lineups. In Experiment 2, the 2-HT eyewitness identification model was used to estimate biased selection of the suspect directly from eyewitness identification decisions. As a first step, we deviated from the recommended standard procedure of the eyewitness task to make the eyewitness task as similar as possible to a mock-witness task. Specifically, lineup rejections were discouraged and participants were alerted that a photograph might stand out from the other photographs in the lineup. When these instructions were used—that are highly unusual for the eyewitness task but typical for the mock-witness task—, the model’s biased-suspect-selection parameter *b* was enhanced in morphed simultaneous lineups in comparison to non-morphed simultaneous lineups, consistent with the measures of unfairness in the mock-witness task. Under these circumstances, using morphed fillers in simultaneous lineups thus lead to the biased selection of the suspects irrespective of their guilt. Based only on the results of Experiments 1 and 2 one may thus be tempted to conclude that the police must stop using morphing techniques to digitally manipulate filler photographs when the lineups are presented in the simultaneous format.

However, in Experiment 3, in which the pre-lineup instructions did not discourage participants from rejecting the lineup, the difference in the biased-suspect-selection parameter *b* between simultaneous morphed and non-morphed lineups was numerically reduced in comparison to Experiment 2 and no longer statistically significant. The difference in biased suspect selection between simultaneous morphed and non-morphed lineups was even completely absent in Experiment 4 in which the pre-lineup instructions did not discourage lineup rejections and did not alert participants that a photograph might stand out from the other photographs in the lineup. This situation most closely corresponds to the standard eyewitness task. The fact that the morphing manipulation did not affect eyewitness identification decisions in the standard eyewitness task contradicts the conclusion that would have to be drawn from the mock-witness data (Experiment 1) and the data obtained in a variant of the eyewitness task that closely mimicked the mock-witness task (Experiment 2). Similar contradictions between mock-witness data and eyewitness results have been reported in other studies^3,10,48^. Together, these results support the assumption that mock-witness choices may not be a good basis for drawing conclusions about eyewitness identification decisions^21^.

Given that the model-based analysis did not yield signs of a morphing unfairness when lineup rejections were not discouraged and participants were not alerted that a photograph might stand out from the other photographs in the lineup, it is possible to assume that these two procedural differences between the mock-witness task and the typical eyewitness task are two major reasons as to why mock-witness choices fail to align with eyewitness identification decisions in the standard eyewitness task: First, whereas mock witnesses are typically required to choose a lineup member, eyewitnesses may choose to reject the lineup. Second, mock witnesses are made aware of the possibility that one lineup member might stand out from the others. Eyewitnesses, in contrast, must make a memory-based identification decision by matching each individual face to their memory representation of the culprit in order to be able to decide whether the culprit is in the lineup. Therefore, the unfairness of the lineup is overestimated in the mock-witness task in comparison to the standard eyewitness task.

Of course, the mock-witness task remains a valuable tool in actual criminal cases, that is, in the situation for which the task has been developed originally, as has been pointed out by Quigley-McBride and Wells^20^. This is so simply because there is currently no better alternative for assessing lineup fairness in practice where the goal is to ensure that a lineup is fair before it is presented to real witnesses. However, as the data presented here have shown, results obtained with mock witnesses may well differ from those obtained with eyewitnesses and thus should be used with caution. In lineup research, in contrast, a measurement model should be used which allows determining whether or not a lineup is unfair in the eyewitness identification situation proper. Otherwise, researchers may draw incorrect conclusions based on invalid fairness assessment procedures, which could lead practitioners to discard appropriate techniques for lineup construction. For instance, here we have shown that morphing artifacts affect mock-witness choices in simultaneous lineups. Such results may well lead policy makers to ban the morphing of photographs for lineup presentation and to eliminate this technique from the set of techniques the police is allowed to use in order to construct lineups. However, as we have shown, the same morphing artifacts that affect choices in situations in which participants have received instructions that are typical of the mock-witness task (Experiments 1 and 2) need not affect eyewitness identification decisions in a typical eyewitness task (Experiment 4). Given these results, there seems to be no reason to ban the morphing of photographs when constructing photo lineups, provided it can be ensured that witnesses receive standard lineup instructions and do not feel pressured to make an identification.

We included both simultaneous and sequential lineups in the present series of experiments because the 2-HT eyewitness identification model is a tool for measuring lineup fairness in both types of lineup formats^32^. This is a distinguishing feature of the present model given that previous research has shown that the mock-witness task is of limited use in estimating lineup fairness in sequential lineups^21,48^. It has previously been shown that unfair simultaneous lineups led to more identifications of innocent suspects than unfair sequential lineups, suggesting that sequential lineups provide more protection for the innocent suspect when the lineup is unfair^3,51,52^. This conclusion is supported by the findings reported here. The results of Experiments 2, 3 and 4 consistently showed no effect of the morphing manipulation on biased suspect selection in sequential lineups, even when the instructions closely resembled those of a mock-witness task. When lineup identifications are made under conditions that do not qualify as best practices—that is, when lineups are unfair and instructions encourage non-memory-based decisions—, sequential lineups provide some protection against unfairness in comparison to simultaneous lineups.

For quite some time, another advantage of sequential lineups seemed to be that, compared with simultaneous lineups, sequential lineups have often been found to be associated with a higher diagnosticity ratio^69^—that is, a higher ratio of the proportion of correct culprit identifications to the proportion of false innocent-suspect identifications^70,71^. This result seemed to indicate that sequential lineups perform better than simultaneous lineups when the goal is to separate culprits from innocent suspects. However, it has been argued that the diagnosticity ratio is an inadequate measure of lineup performance because it confounds the ability to distinguish between a culprit and an innocent suspect with response bias [e.g.,^72^]. Receiver Operating Characteristic (ROC) analyses do not have this problem and have shown either that simultaneous lineups perform better than sequential lineups^23,49,59,73,74^ or that sequential and simultaneous lineups perform equally well^55,75–77^. ROC analyses are said to have the advantage of delivering a performance measure that is not confounded by response bias^23^. However, ROC analyses focussing on the partial area under the curve—that have become commonplace in lineup research—are based on the proportion of correct culprit identifications and false innocent-suspect identifications, as a consequence of which they do not exploit the information contained in filler identifications and lineup rejections separately; these data categories are combined based on the reasoning that both filler identifications and lineup rejections have no legal consequences [^4,78^, but see^79^ for an interesting suggestion on how to create a full ROC based on the full range of response categories]. However, there is information to be gained when these two response categories are analyzed separately. For instance, a filler identification in a culprit-absent lineup is an error. A lineup rejection in a culprit-absent lineup is a correct response. Obviously, many such filler identifications and few lineup rejections indicate bad performance, whereas few such filler identifications and many lineup rejections indicate good performance. The 2-HT eyewitness identification model used here exploits this information in that it takes into account the full range of data categories available from lineup procedures (see Fig. 1). In doing so, the model provides measures for four types of cognitive processes of which we have so far focused on the process of biased suspect selection (represented by parameter *b*) exclusively. We would now like to focus on the process of culprit-presence detection represented by parameter *dP*. An advantage of the 2-HT eyewitness identification model is that parameter *dP* is not confounded with lineup fairness^32,34^, that is, parameter *dP* is a pure measure of culprit-presence detection even in unfair lineups. In the model-based analyses reported here, the estimates of parameter *dP* were consistently higher in the simultaneous lineup conditions than in the sequential lineup conditions (see Table 2). This difference was significant in Experiment 2, ∆*G*^2^(1) = 8.25, *p* = 0.004, *w* = 0.05, and Experiment 4, ∆*G*^2^(1) = 4.80, *p* = 0.028, *w* = 0.04, but not in Experiment 3, ∆*G*^2^(1) = 3.78, *p* = 0.052, *w* = 0.04. A small superiority of simultaneous over sequential lineups was also found by Winter et al.^32^ when applying the 2-HT eyewitness identification model to both simultaneous and sequential lineups. This pattern in the results based on the 2-HT eyewitness identification model is in good agreement with the results of ROC-based analyses in which a superiority of simultaneous over sequential lineups was sometimes found [e.g.,^23,49,59,73,74^] but not always [e.g.,^55,75–77^].

Parallel to the results of Winter et al.^32^, we also found a consistently higher probability of guessing-based selection (captured by parameter *g*) in sequential lineups in comparison to simultaneous lineups in Experiments 2, 3 and 4 (see Table 2). Note that this general pattern is already evident from surface-level data: The rate of identifications was consistently higher in the sequential lineup conditions than in the simultaneous lineup conditions (0.81 vs. 0.67 in Experiment 2, 0.78 vs. 0.58 in Experiment 3 and 0.76 vs. 0.55 in Experiment 4). At first glance, this may seem unexpected given that previous research has indicated that sequential lineups are associated with more conservative responding than simultaneous lineups [e.g.,^75^]. However, in contrast to many previous studies, we did not inform our participants in the sequential lineup conditions that only their first “yes” response counts. Instead, we explicitly followed standard police protocols^24,50,65^ and the original protocol outlined by Lindsay and Wells^80^ and continued the presentation of the sequential lineup even after an early positive response; only the participant’s final decision was coded as their identification decision. This differs from the first-yes-counts protocol that is typically used with sequential lineups in eyewitness research. Horry et al.^65^ have shown that this first-yes-counts protocol systematically reduces suspect identifications and increases lineup rejections by discouraging participants from guessing. These results are easily explained: When only the first “yes” response counts, eyewitnesses may shy away from ‘using up’ their only identification response too early in the sequence because they do not know whether there will be a better alternative later in sequence. This will necessarily lead to conservative responding. In contrast, the (more realistic) lineup protocol that has been used here can be expected to produce relatively liberal responding and thus a higher prevalence of guessing-based selections among lineup members in the sequential lineup. However, this rather interesting aspect of the present study has to be further addressed in future experiments.

Recently, Quigley-McBride and Wells^20^ have proposed an alternative method to measure lineup fairness directly from actual eyewitness data. Specifically, they have recommend calculating the *resultant* lineup fairness based on the innocent-suspect identifications and filler identifications in culprit-absent lineups. Given that it seems interesting to compare these resultant lineup-fairness measures with the biased-suspect-selection parameter *b* of the 2-HT eyewitness identification model, we calculated the average resultant proportion of suspect selections (i.e., innocent-suspect identifications ÷ [innocent-suspect identifications + filler identifications in culprit-absent lineups]) and the average resultant Tredoux’s *E* for Experiments 2, 3 and 4. Note that these calculations are based only on the identifications in culprit-absent lineups whereas the 2-HT eyewitness identification model takes into account all data of both culprit-present lineups and culprit-absent lineups. As a result of being based on a reduced data set, we may expect more variability in the values calculated for the resultant-lineup fairness measures. Still, the resultant proportions of suspect selections reflect the unfairness of morphed opposed to non-morphed lineups in a way that is largely parallel to that of the biased-suspect-selection parameter *b* in the present series of experiments (0.37 vs. 0.29, 0.35 vs. 0.26, 0.29 vs. 0.28 for morphed vs. non-morphed simultaneous lineups in Experiments 2, 3 and 4, respectively; 0.19 vs. 0.22, 0.18 vs. 0.21, 0.25 vs. 0.24 for morphed vs. non-morphed sequential lineups in Experiments 2, 3 and 4, respectively). In addition, the resultant proportions of suspect selections reflect the higher unfairness in simultaneous lineups than in sequential lineups. The resultant Tredoux’s *E* was calculated separately for each of the four simultaneous and sequential morphed and non-morphed lineups before an average resultant Tredoux’s *E* was computed for the simultaneous and sequential morphed and non-morphed lineup conditions, as in Experiment 1. The average resultant Tredoux’s *E* was descriptively smaller for morphed lineups than for non-morphed lineups in Experiment 2, but the data pattern is more variable in Experiments 3 and 4 (4.36 vs. 4.56, 4.41 vs. 4.26, 4.75 vs. 4.16 for morphed vs. non-morphed simultaneous lineups in Experiments 2, 3 and 4, respectively; 5.07 vs. 5.39, 5.37 vs. 5.16, 5.22 vs. 5.29 for morphed vs. non-morphed sequential lineups in Experiments 2, 3 and 4, respectively). In all experiments, the resultant Tredoux’s *E* was descriptively smaller in simultaneous lineups than in sequential lineups (more details on the analyses of the resultant lineup-fairness measures and the distribution of eyewitness identification decisions across lineup members are provided in the Open Science Framework repository at https://osf.io/zaybc/).

A limitation of the present research is that a cross-experiment comparison was used to demonstrate that morphing artifacts cause unfairness in an anomalous identification situation—comparable to that of mock witnesses—but do not enhance biased suspect selection in a standard eyewitness task. Future research could extend the present research by performing a within-experiment comparison to examine more directly how the morphing effect on biased suspect selection interacts with the different lineup conditions. Another limitation of the present study is that only one of the two possible types of mock-witness tasks was used here. Participants were asked to choose the person who stands out from the other lineup members. In another variant of the mock-witness task, participants are provided with a description of the culprit as the basis for their choice [e.g.,^11,12^]. Given that it cannot be taken for granted that the search processes are the same for these two different types of mock-witness tasks, future research should focus on whether the same conclusions can be obtained with the description-based mock-witness task.

## Conclusion

Lineup fairness is a critical factor affecting the likelihood of misidentifications, yet there is surprisingly little research on how to determine the fairness of lineups. Traditionally, researchers have relied on the mock-witness task to evaluate lineup fairness^10^ although this method has been criticized based on the fact that the task of mock witnesses differs from that of eyewitnesses [e.g.,^21^]. The present series of experiments not only demonstrates that those who had questioned the usefulness of the mock-witness task^10,15,20,21,48^ were correct but also sheds light on the crucial differences between the mock-witness task and the eyewitness task that are responsible for the divergent effects. While the mock-witness task showed that morphed lineups were more unfair than non-morphed lineups, the morphing manipulation did not affect eyewitness identification decisions in a typical lineup procedure. This discrepancy was due to two task differences: First, unlike eyewitnesses, mock witnesses are not allowed to reject lineups. Second, mock witnesses are made aware of the possibility that one lineup member might stand out from the others. In contrast, eyewitnesses must match each lineup member to their memory representation of the culprit. In lineup research, it therefore seems desirable to measure lineup fairness directly from eyewitness data using a measurement model such as the 2-HT eyewitness identification model rather than to rely on mock-witness-based measures.

## Data Availability

All raw data analyzed during this study are available in the manuscript or in the Open Science Framework repository (https://osf.io/zaybc/).
